# At the threshold of symbiosis: the genome of obligately endosymbiotic ‘*Candidatus* Nebulobacter yamunensis’ is almost indistinguishable from that of a cultivable strain

**DOI:** 10.1099/mgen.0.000909

**Published:** 2022-12-14

**Authors:** Daniele Giannotti, Vittorio Boscaro, Filip Husnik, Claudia Vannini, Patrick J. Keeling

**Affiliations:** ^1^​ Department of Biology, University of Pisa, Pisa, Italy; ^2^​ Department of Botany, University of British Columbia, Vancouver, Canada; ^3^​ Okinawa Institute of Science and Technology, Okinawa, Japan

**Keywords:** comparative genomics, endosymbiosis, *Euplotes*, *Fastidiosibacter*, genome evolution, intracellular symbionts

## Abstract

Comparing obligate endosymbionts with their free-living relatives is a powerful approach to investigate the evolution of symbioses, and it has led to the identification of several genomic traits consistently associated with the establishment of symbiosis. ‘*Candidatus* Nebulobacter yamunensis’ is an obligate bacterial endosymbiont of the ciliate *Euplotes* that seemingly depends on its host for survival. A subsequently characterized bacterial strain with an identical 16S rRNA gene sequence, named *Fastidiosibacter lacustris *, can instead be maintained in pure culture. We analysed the genomes of ‘*Candidatus* Nebulobacter’ and *Fastidiosibacter* seeking to identify key differences between their functional traits and genomic structure that might shed light on a recent transition to obligate endosymbiosis. Surprisingly, we found almost no such differences: the two genomes share a high level of sequence identity, the same overall structure, and largely overlapping sets of genes. The similarities between the genomes of the two strains are at odds with their different ecological niches, confirmed here with a parallel growth experiment. Although other pairs of closely related symbiotic/free-living bacteria have been compared in the past, ‘*Candidatus* Nebulobacter’ and *Fastidiosibacter* represent an extreme example proving that a small number of (unknown) factors might play a pivotal role in the earliest stages of obligate endosymbiosis establishment.

## Data Summary

Supporting external data were collected for the genomes of *Fastidiosibacter lacustris* strain SYSU HZH-2^T^ (accession no. QLIR00000000) and ‘*Candidatus* Cyrtobacter zanobii’ (accession no. JAJBJB000000000). The authors confirm all supporting data, code and protocols have been provided within the article or through supplementary data files.

**Impact Statement**

We compared the genomes of two extremely close relatives, the bacteria ‘*Ca*. Nebulobacter yamunensis’ and *Fastidiosibacter lacustris *, which are respectively known as an obligate intracellular symbiont and a free-living, cultivable bacterium. While similar comparisons have been extensively used in the past to identify key molecular differences which underlie separate ecological niches, in this system we found virtually none. The host-restricted strain does not show any of the expected molecular signatures of an obligate symbiont, suggesting that subtle changes that are difficult to pinpoint by simply looking at genome features may have consequential biological effects during the very early stages of the transition from free-living bacterium to obligate endosymbiont.

## Introduction

**Fig. 1.**
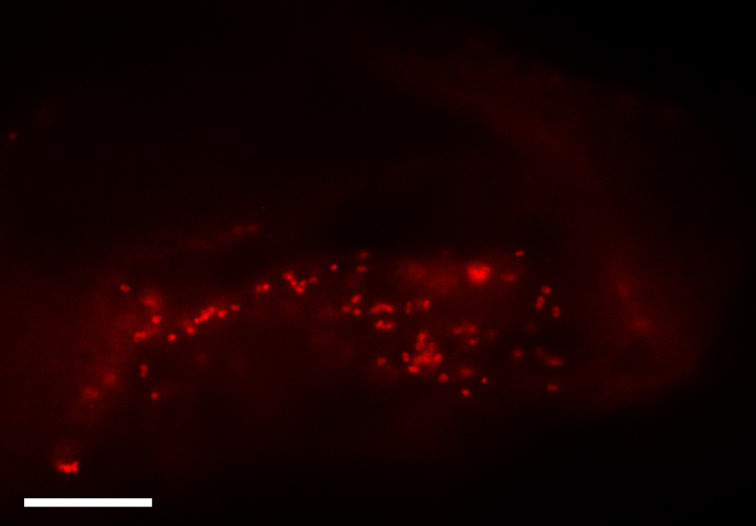
Fluorescence *in situ* hybridization on *Euplotes aediculatus* Eae1. Fluorescent signals from species-specific ‘*Ca*. Nebulobacter yamunensis’ probe NebProb203 [10] highlights the presence of the symbiont within the host cytoplasm. Bar length corresponds to 10 µm.

Intracellular bacterial symbionts, or ‘endosymbionts’, are commonly found associated with a wide variety of hosts, but are largely investigated only in arthropods [1, 2] and a few other macroorganisms [3–5]. Most of the biological diversity of these symbiotic systems is actually found in the smallest of hosts: microbial eukaryotes (protists) [6]. Prokaryote-protist symbioses are especially well-known in ciliates [7], with the widespread genus *Euplotes* emerging as a model system that hosts many symbionts with unknown (but sometimes essential) roles [8, 9]. ‘*Candidatus* Nebulobacter yamunensis’ (* Thiotrichales *, *Gammaproteobacteria *) is one of the so-called ‘accessory’ symbionts of *Euplotes*, inhabiting the cytoplasm of *Euplotes aediculatus* (Fig. 1) [8, 10, 11] from geographical areas as distant as Italy and India. Like other accessory symbionts, ‘*Ca*. Nebulobacter’s’ association with *Euplotes* appears stable under laboratory conditions, even if the bacterium is not proven to be essential for the host like the more well-known *Polynucleobacter* [12]. The reverse is not true: ‘*Ca*. Nebulobacter’ seemingly cannot survive outside its host [10], which is also in line with most symbionts of *Euplotes*. ‘*Ca*. Nebulobacter’ however stands out among accessory symbionts because it does not fall within any clade of specialized intracellular bacteria, such as *Rickettsiales* or *Holosporales* (* Alphaproteobacteria *), which are known for their reduced genomes and infectious features (one representative of these more ubiquitous symbionts, ‘*Ca*. Cyrtobacter zanobii’, is curiously always detected co-occurring with ‘*Ca*. Nebulobacter’ [8]). The closest relatives of ‘*Ca*. Nebulobacter’ are free-living, and one in particular, *Fastidiosibacter lacustris* [13], shares an identical 16S rRNA gene sequence with it.

Very close symbiotic/free-living pairs of organisms are invaluable since the most common approach to investigating changes related to the onset of symbiosis is to compare symbionts with free-living relatives. The longer the time since their divergence, the noisier such data become, which is what makes genera with very close symbiont/free-living pairs such as *Polynucleobacter* [14, 15], *Serratia* [16], and *Sodalis* [17, 18] such important model systems.

We sequenced the genome of the host-restricted ‘*Ca*. Nebulobacter yamunensis’ and compared it with that of the free-living *Fastidiosibacter lacustris* [13] to see if key differences related to the transition to endosymbiosis could be observed. Instead, we found the two genomes to show extreme molecular and functional similarities, despite their apparently contrasting lifestyles, which we confirmed with a new attempt to cultivate both organisms in parallel. Overall, these two bacteria display completely different ecologies without any of the corresponding genomic features that are almost universally observed in other endosymbiotic systems, however recent. This intriguing puzzle may open a new window into understanding the earliest stages in the evolution of intracellular symbioses.

## Methods

### Data collection and genomic analyses

High-throughput sequencing data for ‘*Ca*. Nebulobacter yamunensis’ were obtained as described by Boscaro *et al*. [8] from the metagenome of *Euplotes aediculatus* strain Eae1. Assembly and binning were carried out following Giannotti *et al*. [19], gathering all contigs assigned to *Thiotrichales* in one genomic bin. The contigs were also plotted according to their G+C content and sequencing coverage using the ‘blobplot’ blobtools v1.0 command [20] to distinguish clusters of taxonomically assigned sequences; their maximum and minimum values were used as filters to recover additional unassigned contigs included within these ranges and incorporate them into the final genome. All contigs shorter than 500 bp were discarded.

Genomes belonging to *Fastidiosibacter lacustris* strain SYSU HZH-2^T^ (=NBRC 112274^T^) [13] (accession: QLIR00000000) and to the other accessory symbiont of *E. aediculatus* Eae1, ‘*Candidatus* Cyrtobacter zanobii’ [19] (accession: JAJBJB000000000), were re-analysed in the same way as the newly obtained genome for consistency. The functional annotation of all three genomes was performed with Prokka v1.12 [21], then Artemis v18.1.0 [22] was used to browse through annotations. Putative pseudogenes were identified with Pseudofinder v1.1.0 [23]. A bi-directional best-hit blast against prokaryotic references was performed on the annotated genomes using the KEGG Automatic Annotation Server (KAAS) platform [24] to predict the metabolic and functional traits of the analysed microbes.

Further analyses were specifically performed to compare ‘*Ca*. Nebulobacter yamunensis’ and *F. lacustris* genomes: OrthoFinder v2.3.3 [25] was used to categorize genes and pseudogenes into orthologous groups; NUCleotide MUMmer (NUCmer) v4.0.0beta2 [26] and MAUVE v2.4.0 [27] were employed for large scale pair-wise alignments; the online Average Nucleotide Identity (ANI) calculator (www.ezbiocloud.net/tools/ani) powered by the OrthoANIu algorithm [28] was used to compute their ANI value. The MacsyFinder-based detection tool TXSScan [29] was employed at the Pasteur’s Galaxy web platform (galaxy.pasteur.fr) [30, 31] to screen the genomes for genes involved in bacterial secretion systems. ISfinder (www-is.biotoul.fr) [32] was used to detect putative insertion sequences.

### Experimental procedure for the cultivation of ‘*Ca*. Nebulobacter yamunensis’

A buffered charcoal yeast extract (BCYE) growth medium supplemented with l-cysteine HCl was prepared according to the recipe provided in the Japanese National Institute of Technology and Evaluation (NITE) biological resource centre (NBRC) online catalogue (www.nite.go.jp/nbrc/catalogue/) for medium no. 1366, the same used for the *F. lacustris* type strain stored in NBRC (no. 112274).

*Euplotes* cells were cultivated and maintained under controlled laboratory conditions as previously described [33]. Because cell count and viability of *E. aediculatus* strain Eae1 were incompatible with the procedure at the time, a monoclonal strain of the same species, collected in the same geographic area and harbouring the same accessory symbionts, namely Eae6 [8], was used instead. A culture aliquot was preliminarily treated with chloramphenicol (0.2 mg ml^−1^) overnight to minimize bacterial contamination without affecting the symbiont [14]. Ciliate cells were washed and concentrated through a series of centrifugations, then lysed through sonication (460 Hz; 1 h; room temperature). To replicate the conditions described by Xiao *et al*. [13] in cultivating *F. lacustris *, the lysate was then centrifuged (7000 ***g***, 10 min) and the pellet resuspended in HCl-KCl buffer solution (1 : 9 v/v, pH ~2) for at least 5 min, prior to its inoculation on the BCYE agar medium under sterile conditions. The cultures were incubated at 37 °C under increased (5%) CO_2_ levels (in a NAPCO Series 5400 CO_2_ incubator). A second aliquot from the Eae6 cell lysate was concentrated and fixed in 4 % formaldehyde in PBS for 10 min and prepared for fluorescence *in situ* hybridization (FISH) following the procedure in Vannini *et al*. [12]. Species-specific probe NebProb203 [10] was used to test the lysate for the presence of ‘*Ca*. Nebulobacter yamunensis.’ As positive control, *F. lacustris* strain NBRC 112274 was ordered from NBRC and also inoculated on the BCYE agar medium, and incubated under the same conditions used for ‘*Ca*. Nebulobacter’.

Bacterial growth was checked on agar plates once every day throughout the first week after inoculation, once every 2 days in the second week, and finally once a week (16 total checks). Visible colonies were collected and processed as follows. First, colonies were suspended in two PBS aliquots: total DNA was extracted from the former (NucleoSpin Plant II DNA extraction kit, Macherey-Nagel, modified extraction protocol); the second aliquot was fixed in 4 % formaldehyde in PBS for 10 min and used for FISH. PCRs were performed on the extracted DNA samples for the clonal amplification of the 16S rRNA gene sequences using universal primers for eubacteria (both modified from Lane [34]): 27F (5′-AGA GTT TGA TYM TGG CTC AG-3′) and 1492R (5′-GGN WAC CTT GTT ACG ACT T-3′). Annealing temperature was set at 50 °C for 30 s, followed by 2 min of extension, for 35 cycles. Amplicons were sequenced with the Sanger method and BLASTn was used for sequence identification.

### Data availability

The genome sequence of ‘*Candidatus* Nebulobacter yamunensis’ has been deposited at DDBJ/ENA/GenBank under the accession JAMBMW000000000.

## Results and discussion

### No typical signatures of obligate symbiosis in the genome of ‘*Ca*. Nebulobacter yamunensis’

A 2.1 Mbp-long draft genome was assembled for ‘*Ca*. Nebulobacter yamunensis’ from the metagenome of *Euplotes aediculatus* strain Eae1, including 1986 predicted coding sequences (191 of which are potentially pseudogenes). The general characteristics of the genome are reported in Table 1.

**Table 1.** General genomic features and assembly statistics of *‘Ca*. Nebulobacter yamunensis’ and *Fastidiosibacter lacustris *. “Completeness, %” refers to the values calculated by BUSCO [49]; for insertion sequences, only hits with a bit-score higher than 500 were selected. n/a, not applicable; ORFs, open reading frames

	‘*Ca.* Nebulobacter yamunensis’	* Fastidiosibacter lacustris *
Host species	***Euplotes aediculatus* (ciliate)**	n/a (free-living)
Genome size, bp	**2 098 416**	2 068 591
Contigs, no.	**71**	62
Contigs longer than 1000 bp, no.	**56**	51
Longest contig, bp	**222 531**	256 557
N50, bp	**89 098**	89 533
Average coverage	**46** ×	100 ×
Completeness, %	**97.6**	99.2
G+C content, %	**36.57**	36.33
Total ORF, no.	**1986**	1914
of which, predicted pseudogenes, no.	**191**	129
Coding density, %	**83.2**	83.5
Average gene length, bp	**934**	942
tRNA-coding genes, no.	**38**	38
Insertion sequences, no.	**25**	25
Accession number	**JAMBMW000000000**	QLIR00000000
Reference	**This study**	Xiao et al 2018

**Fig. 2.**
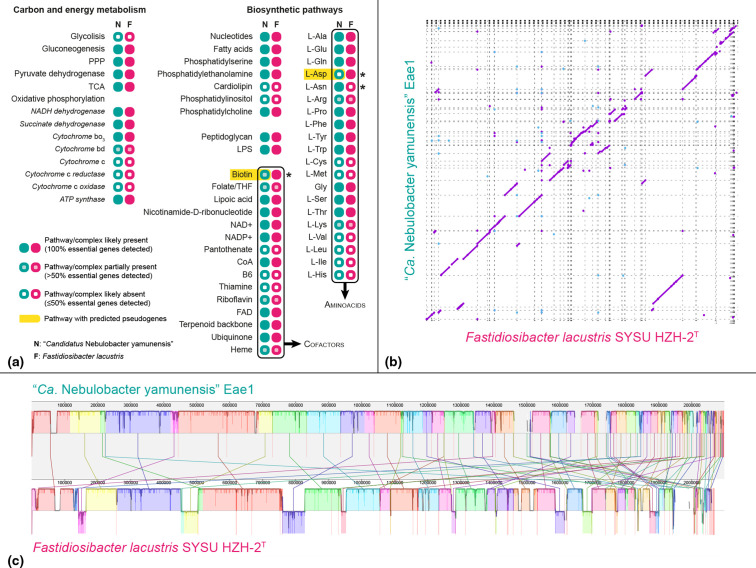
Comparative analyses of ‘*Ca*. Nebulobacter yamunensis’ and *Fastidiosibacter lacustris* genomes. (a): selected predicted metabolic functions (see also Table S1). (b): NUCmer pairwise alignment dotplot: purple - long aligned single-copy regions, blue - repeats (mostly at contig ends); contigs are delimited by dashed lines. (c): MAUVE pairwise alignment; contigs are delimited by thin red vertical lines.

In spite of its relatively small genome size, the reconstruction of ‘*Ca*. Nebulobacter yamunensis’ functional and metabolic traits revealed little depletion (Fig. 2a, Table S1, available in the online version of this article), especially compared with the extensive metabolic impairment reported for other essential [15] and accessory [19] symbionts of *Euplotes*. Nevertheless, while the central carbon/energy metabolism is virtually complete, the lack of the phosphofructokinase-1 gene suggests that the Embden-Meyerhof-Parnas glycolytic pathway is absent, as is also the case with every symbiont of *Euplotes* whose genome has been sequenced to date [9, 14, 15, 19, 35–37]. Complete biosynthetic pathways were also identified for purines, pyrimidines, fatty acids, glycerophospholipids (except phosphatidyl-inositol and cardiolipin), cell envelope components (peptidoglycan and lipopolysaccharides), and most l-amino acids. Exceptions include l-methionine and l-cysteine, where at least half the biosynthetic pathway-specific genes are missing. Only one (shared) gene, *ilvE*, was detected for the l-valine, l-leucine, and l-isoleucine pathways, while none was retrieved for l-histidine biosynthesis. Finally, l-aspartate biosynthesis may be absent due to the pseudogenization of the aspartate aminotransferase gene (Table S2). Co-factor biosynthesis is also mostly intact, with only the pantothenate, pyridoxine, and thiamine pathways likely absent. No corresponding transporter was annotated, suggesting that these co-factors are either not strictly required, or are imported from the host using non-specific (or otherwise annotated) transporters. Corroborating the idea that ‘*Ca*. Nebulobacter’ is an unusually self-sustained symbiont, previous ultrastructural studies showed that stress conditions on the *Euplotes* host have a more conspicuous effect on its essential symbiont, *Polynucleobacter *, than on ‘*Ca*. Nebulobacter’ [11].

An oddly complementary pattern was found between the transporter sets of ‘*Ca*. N. yamunensis’ and the co-occurring accessory symbiont ‘*Candidatus* Cyrtobacter zanobii’ (* Rickettsiales *) [19] (Table S3). For instance, all subunits for ribose (*rbsABC*) and putrescine (*potFGHI*) transporters appear only in ‘*Ca*. Nebulobacter’, while none was detected for ‘*Ca*. Cyrtobacter’. Conversely, malate (*yflS*) and riboflavin (*ribN*) transporters are exclusively found in ‘*Ca*. Cyrtobacter’. In the absence of other clear signs of co-dependency, this pattern alone is unlikely to be a result of co-evolution. Rather, it might be suggestive of a form of niche separation in which the coexistence of ‘*Ca*. Nebulobacter yamunensis’ and ‘*Ca*. Cyrtobacter zanobii’ is facilitated by depleting different resources from the host cytoplasm. Analogous patterns of symbiont co-existence have been previously investigated in bacteriocyte-sharing bacteria in whiteflies [38].

### High similarities in sequence and functional features between an obligately symbiotic and a free-living bacterium

The 16S rRNA gene sequence extracted from the ‘*Ca*. Nebulobacter yamunensis’ genome is identical to those of other known symbiotic ‘*Ca*. Nebulobacter’ strains, as well as that of the free-living *Fastidiosibacter lacustris* strain SYSU HZH-2^T^ (=NBRC 112274^T^) [13]. On a genomic scale, the average nucleotide identity (ANI) between the genomes of ‘*Ca*. Nebulobacter yamunensis’ and *F. lacustris* is 99.38 % (avg. aligned length=1 417 738 bp; genome coverage of 68.67 and 69.64% respectively), well above the conventional 95 % threshold for bacterial species. Pairwise alignments between the two assemblies confirmed their high degree of sequence similarity (Fig. 2b) and synteny (Fig. 2c). Even their overall genomic characteristics appear extremely alike, and many (129) ORFs were predicted to be pseudogenes in *Fastidiosibacter* as well (Table 1); in fact, 79 pseudogenes are shared by both bacteria investigated here and hence likely predate their divergence, rather than being linked to their ecological differences (Table S2). At least 1665 orthologous sequences are shared between the two genomes; among the remaining coding sequences unique either to ‘*Ca*. Nebulobacter yamunensis’ (278) or *F. lacustris* (207), the vast majority encode for unknown hypothetical proteins (82.4 and 73.9% respectively; Table S4). The aforementioned metabolic traits described for the symbiotic ‘*Ca*. Nebulobacter’ are almost identical in the free-living *Fastidiosibacter* (Fig. 2a, Table S1). Minor differences, such as potentially incomplete synthetic pathways for aspartate and biotin (due to pseudogenization of the aspartate aminotransferase gene and *bioF* respectively) in ‘*Ca*. Nebulobacter yamunensis’ (Fig. 2a), do not reflect the expected extensive loss-of-function and genome erosion predicted for even the most recently evolved obligate symbionts. In fact, some gene losses seem to have occurred in the free-living, rather than in the symbiotic lineage, as indicated by the lack of the asparagine synthase gene in *Fastidiosibacter *. Comparative analyses on genes involved in secretion systems also provided no clear indication of different life strategies. The lack of genes involved in substrate recruitment (*virD4*) and target attachment (*virB5*) of type IV secretion systems (T4SSs) in *F. lacustris* might speculatively indicate a more relaxed selective pressure in maintaining the functionality of the corresponding structures, which ‘*Ca*. N. yamunensis’ might still use to mediate important interactions with its host. This remains however largely speculative, since virtually nothing is known about the molecular interactions between bacterial symbionts and ciliate hosts. Finally, *F. lacustris* also possesses a larger set of *tra* genes putatively involved in the F-like type IV conjugative system [39]. No proliferation of mobile elements was found in either genome.

Parallel growth experiments under the same conditions gave the predicted different results for ‘*Ca*. N. yamunensis’ and *F. lacustris *. The former, obtained from the lysate of its host (where it was confirmed to be present by FISH), never produced any colonies, whereas the latter did so 3–4 days after the inoculation.

### What does it take to become a symbiont?

‘*Ca*. Nebulobacter yamunensis’ and *Fastidiosibacter lacustris *, contrary to what their names suggest, are closely related strains of the same species, with little to no differentiating genomic features. Nevertheless, one is an obligate endosymbiont found in multiple strains of the ciliate *Euplotes aediculatus*, stable under laboratory culture conditions but unable, or at least very fastidious, to grow outside its host; the other seems instead to be a typical free-living bacterium, growing under relatively simple conditions and surviving in culture collections without the need for a symbiotic partner.

Any interpretation of this scenario comes with some difficulties. Based on the original environmental isolation of *F. lacustris *, it cannot be completely excluded that it was originally harboured by some host, possibly even *Euplotes*. This however would still not explain why *F. lacustris* is able to grow in isolation and ‘*Ca*. Nebulobacter’ is not. ‘*Ca*. Nebulobacter’/* Fastidiosibacter* might both be opportunistic symbionts, like a gammaproteobacterium relative, *Francisella* [40, 41]. However, opportunistic *Francisella* spp. exhibit a broad host range, while ‘*Ca*. Nebulobacter’ is only known from a single *Euplotes* host species. Moreover, members of *Francisella* can usually be grown in isolation [42], so this explanation alone does not account for the differential cultivability of the strains studied here. Based on current data, we provisionally lean towards the simpler interpretation, i.e. that ‘*Ca*. Nebulobacter’ and ‘* Fastidiosibacter lacustris *’ are two lineages whose ecology diverged so recently that none of the changes usually associated with early stages of endosymbiosis are yet apparent in the genome of ‘*Ca*. Nebulobacter’, making them ‘ecological variants’ of the same microbial species. Changes, genomic or otherwise, might well have occurred but remain undetected because we simply don’t know what to look for.

If this is indeed the case, which should be confirmed by assessing phylogeny and trait diversity among multiple free-living (* Fastidiosibacter *) and symbiotic (‘*Ca*. Nebulobacter’) strains, it is a sign of how much more we need to learn about how bacteria transition from a free-living to an endosymbiotic lifestyle – something that is particularly true if we suspect that drastic changes might occur in the context of regulatory networks [43], which are considerably less understood than changes directly affecting protein structures or metabolic pathways. At present, we cannot link any obvious molecular feature to such a fundamental and striking phenotypic difference as the requirement to live inside a host cell for survival. A well-known example of bacteria with marked phenotypic differences despite high genetic similarities is *Shigella/Escherichia coli,* which can display some morphological, biochemical, serological, and pathological differences, but are indistinguishable from a phylogenetic perspective [44, 45]. For symbiotic/free-living pairs, the arthropod-infecting *Serratia symbiotica* encompasses strains ranging from cultivable extracellular parasites to intracellular host-restricted mutualists [16]. However, while the range of their pairwise ANI values approaches 99 % [46], genomes of *S. symbiotica *’s cultivable and host-restricted strains still differ substantially in size and structural organization [16], contrary to ‘*Ca*. Nebulobacter’/* Fastidiosibacter *. The genus *Sodalis* similarly displays a broad ecological diversification [18]. The host-restricted *Sodalis pierantonius* also shares enough of its genomic sequences with the opportunistic non-symbiotic species, *Sodalis praecaptivus,* to exceed the conventional threshold for species delimitation [18], consistently with its recent symbiotic engagement [17]. However, *Sodalis pierantonius* already displays diverging genomic features, such as an explosive proliferation of insertion sequences [17], while no such difference was recorded in the models investigated here. Another ciliate host, *Heterometopus*, also hosts an archaeal endosymbiont which is not dramatically different from its closest known free-living relative [47]. While several closely related free-living/endosymbiotic microorganisms are known, the *‘Ca.* Nebulobacter’*/Fastidiosibacter* pair stands out as laying at the extreme end of the spectrum of examples, and highlights the sometimes-overlooked possibility that, at least in certain cases, the changes required to become an obligate endosymbiont may be very small [48], and perhaps yet unpredictable, despite the apparently cataclysmic nature of the resulting ecological change.

## Supplementary Data

Supplementary material 1

Supplementary material 2

Supplementary material 3

Supplementary material 4
